# Feasibility of wearable camera use to improve the accuracy of dietary assessment among adults

**DOI:** 10.1017/jns.2022.81

**Published:** 2022-09-27

**Authors:** Judith L. Scott, Aswathy Vijayakumar, Jayne V. Woodside, Charlotte E. Neville

**Affiliations:** Centre for Public Health, School of Medicine, Dentistry and Biomedical Sciences, Queen's University Belfast, Grosvenor Road, Belfast BT12 6BJ, UK

**Keywords:** 24-h recall, Dietary assessment, Eating habits, Wearable camera

## Abstract

Traditional methods of dietary assessment are prone to measurement error, with energy intake often under-reported. The 24-h recall is widely used in dietary assessment, however, its reliance on self-report without verification of consumption can result in inaccuracies in true nutrient intake. Wearable cameras may provide a complementary approach to improve self-report accuracy by providing an objective and passive measure of food consumption. The purpose of the present study was to determine whether a wearable camera improves the accuracy of a 24-h recall compared with a 24-h recall alone in twenty adults aged 18–65 years. The study also explored limitations associated with wearable cameras. Participants wore the camera for 1 d and a 24-h recall was then conducted the following day, before and after viewing the camera images. Dietary data were analysed using Nutritics dietary analysis software, while eating habits were assessed by a self-report questionnaire. Energy and nutrient intakes were compared between the recall alone and the camera-assisted recall. Results showed a significant increase in mean energy intake with the camera-assisted recall compared with the recall alone (9677⋅8 ± 2708⋅0 kJ/d *v*. 9304⋅6 ± 2588⋅5 kJ/d, respectively, *P* = 0⋅003). Intakes of carbohydrates, total sugars and saturated fats were also significantly higher with the camera-assisted recall. In terms of challenges, there were occasionally technological issues such as proper positioning of the camera by the participants. In conclusion, reporting of energy and nutrient intake may be enhanced when a traditional method of dietary assessment, the 24-h recall, is assisted by a wearable camera.

## Introduction

Accurate estimation of dietary intake is difficult, with methods commonly employed (e.g. food frequency questionnaires, 24-h recalls and food diaries) each being associated with random and inherent error^(1–3)^. One of the most commonly reported errors in dietary assessment relates to the under- or over-reporting of food intake^(4–6)^. Many dietary assessment methods also tend to be burdensome for both the participant and the researcher^(7)^. Diet, along with many other lifestyle factors, is implicated in disease risk therefore in order to gain a better understanding of how diet is related to the risk of disease we need to ensure that methods used to assess and measure dietary intake are accurate^(8,9)^. The 24-h recall is a commonly used dietary assessment tool which provides a subjective measure of dietary intake^(3,10)^. While the 24-h recall provides a relatively quick and quantifiable assessment of food intake it nonetheless has the potential for error due to the reliance on the memory of the participant^(7,11,12)^. Indeed, previous studies have highlighted that specific foods, particularly snack foods, condiments and beverages, are commonly under-reported in 24-h recall assessments^(12,13)^.

Technology is gradually being incorporated into dietary research as a means of trying to improve the accuracy of dietary assessment^(3,11,14–16)^. A number of electronic devices and technologies have been explored, including portable cameras, mobile phone apps and online tools^(3,14,15,17–20)^. However, many of these methods require extra input and compliance by the respondent in terms of manual activation of the device by the user, remembering to manually take photographs of each episode of eating or adding foods to an app or online webpage^(3,14,15,19,20)^. Research into the use of wearable cameras to enhance dietary recall is of significant interest in public health^(21)^. Preliminary research suggests that the use of technology in the form of a wearable camera may reduce respondent burden of recording dietary intake, providing a more non-invasive and complementary dietary assessment method to enhance dietary recall assessment and reduce under-reporting of energy intake^(22–24)^. Such devices provide passive and objective information regarding food intake and require minimal input from the participant as they are programmed to capture images automatically thereby removing the inherent issue of reporting bias common to traditional dietary assessment methods^(13,25)^.

A number of studies which examined the efficiency of a wearable camera to improve the accuracy of dietary intake assessment reported an increase in accuracy of estimated total energy intake when compared with either a recall alone or when the camera was used alongside another method^(12,22,25–27)^. Shim *et al.* assessed various dietary assessment methods and recognised that using technology along with traditional methods of assessment improved the accuracy of dietary intake^(28)^. Gemming *et al.* similarly demonstrated that wearable cameras may be effective in improving dietary intake accuracy^(12,25)^. While previous studies suggest an improvement in dietary intake assessment with wearable technology, these studies were conducted in specific research populations, such as young athletes, or were conducted in specific age groups^(13,26,27)^. Therefore, evidence for the use and the effectiveness of this type of technology in the general population is limited. Active food photography to aid in portion-size estimation introduces the possibilities of conscious over or under estimation of dietary intake based on social desirability^(29)^. The existing studies on wearable cameras have also failed to examine social settings and the impact of social setting on eating patterns^(13,26,27)^. The aim of the present study was to determine the type of wearable camera most acceptable by individuals for capturing food intake and to evaluate whether or not a wearable camera can enhance the accuracy of a 24-h recall. The present study also examined the usability and practicality of a wearable camera in everyday life as a means of assessing habitual food intake.

## Methods

### Study design

#### Exploratory study to determine the most suitable camera for capturing food intake

**Fig. 1.**
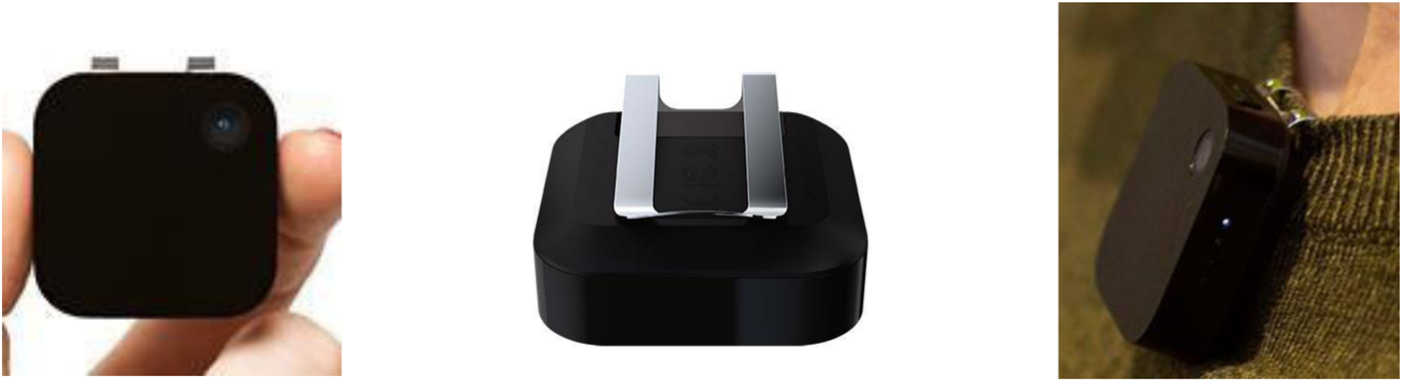
Visual image of the Narrative Clip^(30)^.

An exploratory study was initially conducted with five participants to determine which method of imaging food intake was the most feasible. Each participant was provided with the ‘Autographer’ wearable camera, the ‘Narrative Clip’ wearable camera and a mobile phone camera for capturing food intake. Participants wore each device for 1 d after which they reported back their preferred choice for capturing their food intake throughout the day. The feedback questionnaire was based on the study and asked the participants their preference of (a) device and why – this allowed many different responses to be acquired which was (b) insightful for dietary recall analysis. Of the five participants partaking in the study, three of those stated that the ‘Narrative Clip’ was both the most enjoyable camera to wear and the easiest device to use. The ‘Narrative Clip’ was chosen as the most preferred method, 80 % of the study participants selected this camera. The reasons for preference included its ease of use, the automatic nature of the camera, the discreet size of the device and the fact that it did not disturb daily activities. The ‘Narrative Clip’ is a small, lightweight, automatic 5-megapixel camera which has a large storage capacity for images and a typical battery life of 2 d^(30)^. The camera can be clipped easily onto any clothing, making it effortless to wear (Fig. 1). It also automatically captures images every 30 s throughout the day, therefore capturing camera footage of all food and/or drinks consumed throughout the day.

#### Ethics

This study was conducted according to the guidelines laid down in the Declaration of Helsinki and all procedures involving human subjects were approved by the Research Ethics Committee of the School of Medicine, Dentistry and Biomedical Sciences at Queen's University Belfast (ref 17.54v2). Written informed consent was obtained from all subjects.

#### Recruitment of participants for the ‘Narrative Clip’ study

Based on the findings of the exploratory study, the ‘Narrative Clip’ wearable camera was subsequently used to examine its effectiveness in improving the accuracy of a 24-h recall.

For the purposes of this study, participants were recruited by word of mouth from an urban area of Northern Ireland over a 3-month period. To be eligible for the study, participants had to be aged 18–65 years and in good health. Participants who expressed an interest in the study were screened to assess their suitability for the study and were given both verbal and written information about the study. For the study twenty healthy, free-living volunteers aged 18–65 years were selected.

#### Use of the ‘Narrative Clip’ wearable camera

Following written consent, a convenient day was chosen by each participant to wear the ‘Narrative Clip’ camera from waking in the morning, after washing and dressing, until going to bed. The camera was clipped onto the clothing of the participant. Participants were supplied with verbal and written instructions on how to operate the camera (including battery life and correct placement of the device) and were also reminded that the camera could be removed at any stage during the day if they felt uncomfortable or were in an inappropriate place, for example at the gym or bathroom.

### 24-h dietary recall

The day after wearing the camera, the camera images obtained with the ‘Narrative Clip’ camera were uploaded onto a laptop for viewing. While the images were being uploaded, a 24-h recall interview was conducted with the participant. The 24-h recall consisted of three steps: Step (1) an initial quick list of everything the participant had eaten throughout the day, followed by Step (2) a more in-depth review of food consumption including: type of food eaten, location of where the food was consumed, amount of food, cooking methods used to prepare the food and whether the food was eaten alone or with other people. To improve accuracy, participants were shown photographs of different portion sizes to assist in quantifying the amount of food eaten^(31)^, Step (3) was a final review of food items and amounts recalled.

**Fig. 2.**
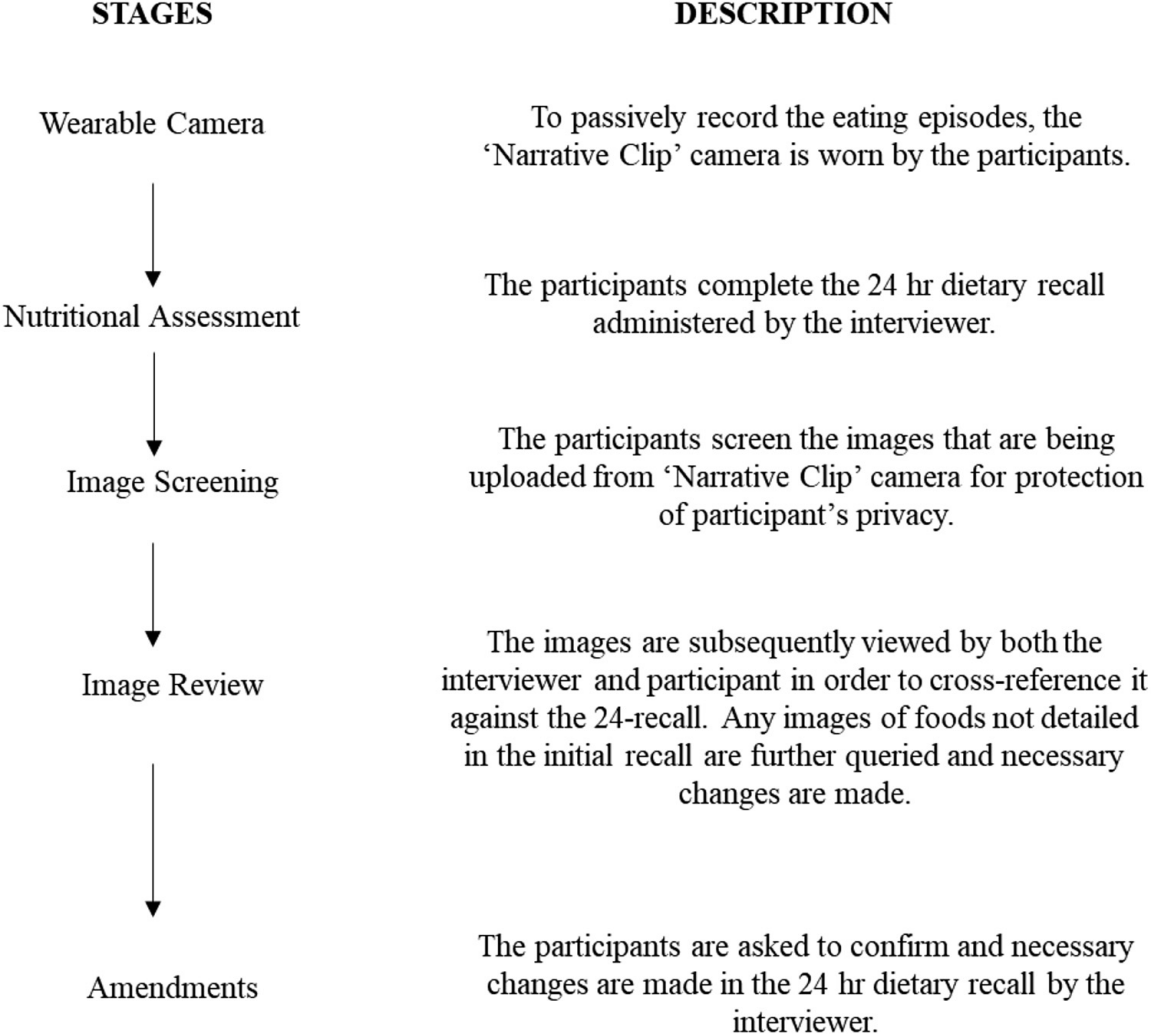
Flow diagram showing the steps involved in conducting the 24-h recall alone followed by the 24-h recall assisted by the Narrative Clip.

On completion of the 24-h recall the participant was asked to screen the images captured by the camera and remove any images they did not wish the researcher to see, thus ensuring that participant privacy was maintained^(32)^. The images were subsequently viewed by both the researcher and participant in order to identify each eating episode and to cross-reference it against the 24-h recall. This enabled the researcher to address any ambiguities within the dietary recall and to confirm, add, remove or modify details of consumption reported in the initial 24-h recall. During this process, the researcher verbally relayed the foods and portion sizes initially recalled. Any images of foods which were not detailed in the initial recall were further queried and any changes made to the initial 24-h recall recorded on a separate recall log, including any change to portion sizes, location of consumption and whether foods were consumed alone or with others. Following completion of the recall, all images were deleted from the laptop in the presence of each participant to ensure privacy. A flow diagram of the 24-h dietary recall method is summarised in Fig. 2.

### Questionnaires

Participants were also asked to complete two questionnaires after wearing the camera: an eating patterns questionnaire and a usability questionnaire. The eating patterns questionnaire included questions relating to employment status, living conditions, eating patterns (home *v*. work, weekday *v*. weekend), education, smoking and alcohol consumption status. Participants were also asked to provide an estimation of their height (to the nearest metre) and weight (to the nearest kilogram). Body mass index (BMI) was subsequently calculated as weight/(height)^2^. The usability questionnaire was used to gain feedback from participants regarding their likes and dislikes about the wearable camera and the feasibility and practicality of using such devices in future dietary assessment studies.

### Statistical analysis

Statistical analyses were conducted using SPSS version 20.0 IBM. Data from participants were only included in the final analysis if they adhered to the study protocol and correctly wore the camera for 1 d. All dietary data were analysed using dietary analysis software (Nutritics, Dublin, Ireland) which generated an output of mean energy and nutrient intake for each participant. The dietary data were entered and analysed as two separate logs, i.e. single 24-h recall versus the camera-assisted recall (i.e. the single 24-h recall plus additional foods/drinks captured by the camera images). The nutrients of interest included energy, protein, total fat, carbohydrates, starch, total sugars, non-starch polysaccharide (NSP), saturated fat (SFA), monounsaturated fat (MUFA), polyunsaturated fat (PUFA), calcium, iron and vitamin C. All dietary data generated from Nutritics were exported to SPSS for statistical analysis. Continuous variables are presented as mean (sd) while categorical variables are presented as *n* (%). Differences in energy and nutrient intake between the recall and camera-assisted recall were assessed using a paired *t*-test. For all analyses, *P* < 0⋅05 was considered statistically significant.

## Results

### Study participants

Participant characteristics are shown in Table 1. Twenty healthy free-living volunteers participated in this study, ten males and ten females with a mean age of 32⋅5 years. All participants completed the full study protocol. Participants had a mean BMI of 25⋅6 (sd 4⋅1). The participants had spent on average 16⋅4 (sd 2⋅6) years in full-time education and the majority were married. All participants, with the exception of one, had never smoked, while the majority were alcohol consumers.

**Table 1.** Lifestyle characteristics of the twenty study participants

Characteristics	*n* (%)
Age (years)	
18–24	8 (40)
25–44	6 (30)
45–65	6 (30)
Sex	
Male	10 (50)
Female	10 (50)
Marital status	
Single	10 (50)
Cohabiting	1 (5)
Married	9 (45)
Employment status	
Full time	10 (50)
Self-employed	2 (10)
Homemaker	1 (5)
Student	7 (35)
Smoking status	
Yes	0 (0)
No	19 (95)
Previous smoker	1 (5)
Consumer of alcohol	
Yes	13 (65)
No	7 (35)
Height (m)	1⋅73 (0⋅14)^a^
Weight (kg)	77⋅3 (19⋅2)^a^
BMI (kg/m^2^)	25⋅6 (4⋅1)^a^
Education (years)	16⋅4 (2⋅6)^a^

BMI, body mass index; sd, standard deviation.

^a^Values are mean (sd).

### Results of the exploratory study

Among the five participants who were part of the exploratory study, all of them reported that the ‘Autographer’ camera was their least favourite to use as the device was intrusive, embarrassing to wear, too large, too noticeable, short battery life, difficult to use and not user-friendly. 80 % of the participants preferred the ‘Narrative Clip’ camera as their favourite method to record dietary intake especially because this was the easiest device to use. The participants also stated that the ‘Narrative Clip’ camera was discreet in nature because of its small size and did not disturb daily activities. Mobile phone was chosen as the favourite camera to use by 20 % of the participants as it did not need to be displayed at all times, adding to the convenience of the device. Two individuals felt that the camera was an invasion of their privacy as it was noticeable by other people and difficult to be worn to work.

### Eating patterns

Eating pattern characteristics of the participants are shown in Table 2. Of the twenty participants, only one participant lived alone, with the remaining nineteen participants living with two or more people. Four participants (20 %) stated that they ate alone, while the majority (*n* 16, 80 %) ate with other people. When asked about food consumption patterns, three participants (15 %) stated that they ate more while at work and fifteen participants (75 %) mentioned they ate more when not at work. Four participants (20 %) noted that they were following a special diet.

**Table 2.** Eating patterns reported by twenty study participants

Eating pattern variable	*n*	(%)
Weekday meals		
Rushed	12	60
Relaxed	7	35
Neither	1	5
Weekend meals		
Rushed	3	15
Relaxed	17	85
Neither	0	0
Time taken to eat meals during weekdays		
Breakfast		
5 min	8	40
10–15 min	11	55
20 min	1	5
Lunch		
5–10 min	8	40
15–20 min	10	50
30–35 min	2	10
Dinner		
0–10 min	4	20
15–20 min	9	45
30–45 min	6	30
60 min	1	5
Time taken to eat meals during weekend		
Breakfast		
0–5 min	4	20
10–15 min	12	60
20 min	2	10
30 min	2	10
Lunch		
5–10 min	6	30
15–20 min	10	50
25–30 min	3	15
45 min	1	5
Dinner		
10–15 min	4	20
20–30 min	14	70
60 min	2	10

sd, standard deviation.

Values represent the mean minutes taken to eat meals based on continuous data.

### Energy and nutrient intakes

**Fig. 3.**
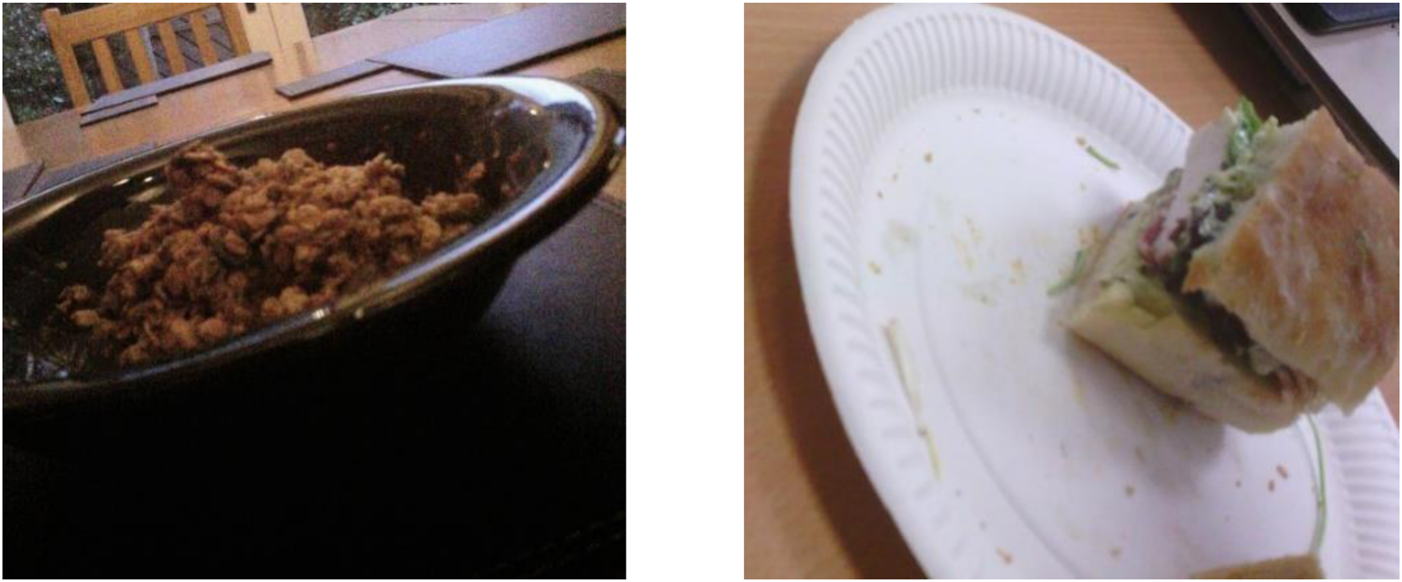
Example of food images captured by the Narrative Clip.

Mean (sd) energy and macronutrient intakes are shown in Table 3. Mean energy intake recorded with the camera-assisted 24-h recall was significantly greater compared with the 24-h recall alone (9677⋅8 ± 2708⋅0 kJ/d *v*. 9304⋅6 ± 2588⋅5 kJ/d, *P* = 0⋅003). Intakes of carbohydrates, total sugars and SFA were also significantly higher with the camera-assisted recall compared with the 24-h recall alone (all *P* < 0⋅05). There were no significant differences in the intakes of vitamin C, calcium, iron, PUFA, MUFA, protein, total fat, starch and NSP between the two methods. Fig. 3 shows some typical images captured by the camera.

**Table 3.** Energy, macronutrient and micronutrient intakes for a standard 24-h dietary recall interview compared to a 24-h recall plus wearable camera

Nutrient	24 h recall	24 h recall + camera	Difference 1	*P*-value
Energy (kcal)	2206⋅5 (609⋅0)	2295⋅7 (637⋅7)	−89⋅2 (118⋅2)	0⋅003
Energy (kJ)	9304⋅6 (2588⋅5)	9677⋅8 (2708⋅0)	−373⋅2 (492⋅9)	0⋅003
Protein (g) Total	98⋅2 (46⋅9)	100⋅5 (46⋅3)	−2⋅3 (7⋅8)	0⋅200
Fat (g)	75⋅4 (29⋅1)	79⋅2 (31⋅3)	−3⋅8 (8⋅8)	0⋅070
Carbohydrate (g)	279⋅7 (96⋅5)	289⋅8 (101⋅1)	−10⋅1 (12⋅8)	0⋅002
Starch (g)	153⋅9 (56⋅5)	156⋅0 (57⋅7)	−2⋅2 (5⋅5)	0⋅090
Sugars (g)	123⋅5 (59⋅6)	130⋅9 (62⋅8)	−7⋅4 (10⋅3)	0⋅004
NSP (g)	13⋅9 (8⋅1)	14⋅1 (8⋅5)	−0⋅21 (0⋅53)	0⋅100
SFA (g)	27⋅7 (16⋅4)	28⋅9 (16⋅5)	−1⋅2 (2⋅2)	0⋅300
MUFA (g)	20⋅1 (10⋅5)	20⋅4 (10⋅9)	−0⋅30 (0⋅96)	0⋅200
PUFA (g)	9⋅4 (5⋅5)	9⋅5 (5⋅7)	−0⋅083 (0⋅41)	0⋅370
Calcium (mg)	831⋅9 (488⋅4)	860⋅1 (495⋅0)	−28⋅2 (72⋅0)	0⋅100
Iron (mg)	9⋅6 (4⋅1)	10⋅1 (5⋅4)	−0⋅49 (2⋅09)	0⋅310
Vitamin C (mg)	114⋅1 (144⋅6)	119⋅0 (146⋅2)	−4⋅9 (21⋅5)	0⋅320

NSP, non-starch polysaccharide; SFA, saturated fat; MUFA, monounsaturated fat; PUFA, polyunsaturated fat; sd, standard deviation.

Values are means (sd).

### Unreported dietary intake

The camera-assisted dietary recall provided an insight into the food items that were misreported during the initial recall. These misreporting errors are shown in Table 4 and varied from over-reporting or under-reporting of dietary intake, and changes in food preparation. These additional misreported foods were categorised into the following groups; hot (tea and coffee) and cold beverages (excluding water due to 0 kJ of energy), dairy, condiments (including spreads, sauces, dips and flavourings), bread and cereal, fruit and vegetables, cooked meats, vitamins and snacks (including biscuits, crisps, sweets, chocolate and ice pops). The camera revealed forty-six misreported items; forty-four items were the result of under-reporting; one item was a change in food preparation and the remaining item was an over-reporting error. Three of the forty-four under-reporting errors were due to water consumption not being reported therefore were not counted. Under-reported items were evenly distributed across the data collection days (weekend *v*. week day). Five participants (25 %) did not make any changes to their 24-h dietary recall after viewing the camera images.

**Table 4.** Additional food items recalled by participants after viewing the wearable camera images

Category	Under-reported	Over-reported	Change in food preparation
Cold beverages	2		
Hot beverages	5		
Bread and cereal	2		1
Condiments	11		
Cooked meats	3		
Dairy	3	1	
Fruit and vegetables	4		
Snacks	9		
Vitamins	1		
Total	41	1	1

Values represent the number of participants.

### Evaluation of the wearable camera

As shown in Table 5, the majority (95 %) of participants stated that they found the wearable camera easy to use. Typical comments included: ‘the camera was easy to clip onto clothes’, ‘lightweight’ and ‘not intrusive’. One participant stated that they ‘forgot the camera was there throughout the day’. Another individual commented that it was difficult to ascertain how much battery charge was remaining in the camera.

**Table 5.** Evaluation of the wearable camera by the participants

Questions asked	*N* (%)		
	Yes	Somewhat	No
Did you find the camera easy to use?	19 (95)	0 (0)	1 (5)
Did you enjoy wearing the camera?	11 (55)	11 (5)	8 (40)
Did you find wearing the camera invasive of privacy?	3 (15)	13 (65)	4 (20)
Were you conscious of wearing the camera?	9 (45)	9 (45)	2 (10)
Did wearing the camera alter your eating habits?	2 (10)	18 (90)	0 (0)

In relation to the enjoyment of wearing the camera, four individuals (20 %) commented that they were unaware of the camera or that wearing the device did not bother them. However, two participants (10 %) stated that the camera made them more conscious of what they ate and that they tended to snack less when wearing the camera. They also noted that they were conscious that everyone could see the device if they were in a public location. While the majority (*n* 13, 65 %) of participants felt that the camera did not invade their privacy, some (*n* 7, 35 %) highlighted that use of the camera while using the bathroom and also in public situations was invasive. Nine participants also stated that they were conscious of the wearable camera throughout the day, especially in terms of whether the camera was positioned correctly to capture all food intake. Two participants also reported that the camera moved throughout the day or physically fell off their clothes. When viewing the images, the researcher also noted that some images were obstructed, for example due to hair, clothing and movement.

In terms of the preferred method of dietary assessment, i.e. wearing a camera, pen and paper method or interview with a researcher (dietary recall), the majority (*n* 10) stated that they preferred the pen and paper method. Only three reported an interview with a researcher, i.e. a dietary recall, as their preferred method, while the remaining seven participants showed preference towards wearing a camera prior to taking part in a dietary recall. Those who preferred the camera referred to the factual nature of the camera, no reliance on memory and no additional work required to wear the device. Those who preferred to write down their food intake (pen and paper method) commented on the ease of this method compared with potential problems which could arise with the camera, e.g. the camera falling off, the battery failing. They also noted that the pen and paper method was non-invasive and that foods consumed are recorded at the time of consumption therefore there was no reliance on memory and less chance of forgetting what they ate. Those (*n* 3) who preferred an interview with a researcher based their opinion on the awareness that clothing may affect camera images captured and the ease of discussing their food intake with an interviewer.

## Discussion

This study highlights that a wearable camera can be beneficial in improving the accuracy of a traditional dietary assessment method, such as the 24-h recall, through the possession of actual intake images captured in real time. Overall, the findings highlighted significant differences between the 24-h recall and the camera-assisted 24-h recall with the wearable camera resulting in significantly higher energy, carbohydrate, total sugars and SFA intakes compared with the 24-h recall alone. The findings showed that snack foods were the main source of under-reporting. Initially, an exploratory study was conducted with five participants to determine which method of imaging food intake was the most feasible and the ‘Narrative Clip’ was chosen as the most preferred method.

Few studies have investigated the use of wearable cameras for dietary assessment purposes^(12,13,17,22,25–27)^. Gemming *et al.* conducted a study in participants aged 18–65 years using another type of wearable camera, Sensecam, to record dietary intake for 2 d with a dietary recall performed on the third day^(25)^. Gemming *et al.* noted a significant increase in mean energy intake along with increases in protein, total fat, SFA and MUFA when the dietary recall was assisted by the camera images^(25)^. Another study using micro-camera located on the ear of the participants also showed that the use of a wearable camera resulted in an increase in mean energy intake along with increases in intakes of protein, carbohydrate and fat intakes^(26)^. Similarly, Chan *et al.* using the ‘Autographer’ wearable camera found that in young adults aged 18–30 years reporting of discretionary snacks was omitted^(17)^. Some studies have used the ‘Narrative Clip’ as the wearable camera. Zhou *et al.* conducted a study where the children aged 9–10⋅9 years wore the ‘Narrative Clip’ for 7 d and performed a 24-h recall at home alone and found that wearable cameras improved accuracy in measuring dietary intake when compared with a 24-h recall alone^(13)^. Their results are similar to those of the current study where the wearable camera resulting in significantly higher energy, carbohydrate and SFA intakes compared with the 24-h recall alone. Additionally, the current study also found that the intake of sugar was significantly higher when compared with the 24-h recall alone. In general, the automated nature of the wearable camera removed the typical inherent reporting bias that is common with traditional dietary assessment methods. The type of foods that were captured by the camera and those reported is noteworthy and thus allows us to identify foods that are commonly under-reported. Thus, the study highlights the benefits of a wearable device for improving the accuracy of the dietary assessment.

The drawbacks with the wearable camera that were observed in this study reflect those observed in previous studies^(21)^. Technological issues were one of the major limitations. Correct positioning of the camera was a challenge in that it if placed incorrectly it resulted in photos of food items being obscured. The photo quality also depended on the position of the participant, i.e. sitting or standing to eat. The nature and feasibility of using a wearable camera in everyday life also has its issues particularly in terms of invasion of privacy and when interacting with the general public or in certain social situations. This was observed on several occasions whereby participants were conscious that they were wearing the device and had to remove the camera, for example in specific occupations or when going into the bank or using the bathroom. Overall though, while invasion of privacy is a potential limitation of the camera, it did not appear to be a major issue in the present study. Reactivity was also affected in that some participants reported that the camera altered their eating habits by making them more conscious of the foods they were eating. This may lead to changes in eating behaviour and thus may falsely capture normal habitual intake. Furthermore, in the present study, the participants only wore the camera for 1 d which likely did not give a true representation of habitual food intake. Moreover, while the camera captures pictures of food eaten it does not provide any additional information regarding the amount of food or portion size. Reviewing the images was time-consuming for both the researcher and participant as there were a lot of duplicated photos due to the frequency of the photos being taken.

A key strength of the present study was the high compliance rate, with all participants providing complete data and wearing the camera as required. This is encouraging and indicates the feasibility of incorporating this type of camera technology into dietary assessment studies. Previous studies have discussed that the use of technology, especially wearable camera may reduce respondent burden of recording dietary intake. The ease of use associated with wearable camera makes it preferable when compared with food diary records, which cause relatively large respondent burden. The use of a wearable camera for examining food intake may also be warranted when assessing dietary intake of individuals with memory recall, for example older adults who may have some degree of cognitive impairment. The present study had a larger sample size than previous studies. Additionally, unlike previous studies, which were conducted in specific population groups (children, young adults, athletes), this study included a diverse age range therefore results could be transferrable to similar population groups^(13,26,27)^. The ‘Narrative Clip’ has not been used widely in previous dietary assessment studies; therefore, this study provides novel evidence for the possibility of this camera being utilised in dietary assessment. The ease of use of this camera, the small size and the discreet nature of the device made it ideally suited for the purposes of this research. This study also gathered data on eating behaviours (household size, eating with others, reported eating speed and time taken to eat) to characterise the settings in which participants recorded their dietary intake using the wearable camera. In the present study, 95 % of the participants lived with two or more people, and 80 % of the participants ate with other people. Furthermore, 85 % of the participants had ‘relaxed’ eating pattern during weekend mealtimes compared with 35 % of the participants having a ‘relaxed’ eating pattern during weekdays. Therefore, this sample had significant diversity in participant eating patterns, yet participants reported no impact on their ability to wear the camera for the duration of the study.

Further research, especially with a larger sample size is required to determine the practicality and usability of the camera over a longer time period and to explore whether wearing the device for a longer time period would impact on dietary intake and eating habits. Additionally, future studies should examine the cost associated with including wearable cameras as part of dietary assessment in large-scale studies. The utilisation of mobile phones for recording dietary intake also requires further research particularly since our initial exploratory study revealed a lack of preference towards a mobile phone for recording dietary intake.

## Conclusion

In conclusion, this study found that the wearable cameras significantly reduced the amount of under-reporting which occurred in the 24-h recall. Based on the exploratory study, the ‘Narrative Clip’ was chosen as the most preferred device as wearable camera. The images captured by the ‘Narrative Clip’ proved to be a useful aid for dietary recall allowing individuals to view consumed foods. Thus, the use of a wearable camera significantly decreased the extent of under-reporting of energy and nutrient intake in this study, thereby highlighting the potential ability of such a device to enhance the accuracy of self-reported dietary assessment methods. The study further characterised factors that potentially influences eating behaviours and found that this diverse sample found the camera acceptable to wear. Studies with larger sample sizes are needed to further examine the feasibility of using wearable cameras along with 24-h recall for collection of dietary data in future studies.
